# Developing Creativity to Enhance Human Potential in Sport: A Wicked Transdisciplinary Challenge

**DOI:** 10.3389/fpsyg.2019.02090

**Published:** 2019-09-13

**Authors:** James Vaughan, Clifford J. Mallett, Keith Davids, Paul Potrac, Maurici A. López-Felip

**Affiliations:** ^1^School of Human Movement and Nutritional Sciences, The University of Queensland, Brisbane, QLD, Australia; ^2^Research and Development Department, AIK Fotboll, Stockholm, Sweden; ^3^Faculty of Sport and Health Sciences, Technical University of Munich, Munich, Germany; ^4^Skill Acquisition Research Theme, Centre for Sports Engineering Research, Sheffield Hallam University, Sheffield, United Kingdom; ^5^Department of Sport, Exercise and Rehabilitation, Northumbria University, Newcastle upon Tyne, United Kingdom; ^6^School of Public Health, Physiotherapy and Sport Science, University College Dublin, Dublin, Ireland; ^7^Department of Psychological Sciences, Center for the Ecological Study of Perception and Action, University of Connecticut, Storrs, CT, United States; ^8^Team Sports Department, Futbol Club Barcelona, Barça Innovation Hub, Sant Joan Despí, Barcelona, Spain

**Keywords:** transdisciplinary research, wicked problems, ecological dynamics, sociocultural constraints, forms of life, non-linear pedagogy, coaching, football

## Abstract

The challenge of developing creativity to enhance human potential is conceptualized as a multifaceted *wicked problem* due to the countless interactions between people and environments that constitute human development, athletic skill, and creative moments. To better comprehend the *inter-relatedness* of ecologies and human behaviors, there have been increasing calls for transdisciplinary approaches and holistic ecological models. In this paper we explore an ecological dynamics rationale for creativity, highlighting the conceptual adjacency of key concepts from transdisciplinarity, dynamic systems theory, ecological psychology and social-cognitive psychology. Our aim is to extend the scope of ecological dynamics and contextualize the application of non-linear pedagogy in sport. Foregrounding the role of sociocultural constraints on creative behaviors, we characterize the athlete-environment system as an ecological niche that arises from, and simultaneously co-creates, a *form of life*. We elaborate the notion that creative moments, skill and more generally talent in sport, are not traits possessed by individuals alone, but rather can be conceived as properties of the athlete-environment system shaped by changing constraints. This re-conceptualization supports a pedagogical approach predicated on notions of athletes and sports teams as complex adaptive systems. In such systems, continuous non-linear interactions between system components support the exploration of fluent and flexibly creative performance solutions by athletes and sports teams. The implications for practice suggest that cultivating a constellation of constraints can facilitate adaptive exploration of novel affordances (opportunities/invitation for action), fostering creative moments and supporting creative development in athletes. Future models or frameworks for practice contend that pedagogies should emerge from, and evolve in, interaction with the sociocultural context in which practitioners and athletes are embedded.

## Introduction

Historically, the boundaries of human potential (across many domains) have tended to be re-configured by serendipitous situations and creative moments. Moments of creativity have advanced our knowledge, re-shaped our lives, altered our understanding, and challenged the perceived limits of what can, and cannot, be achieved (Songca, 2006; Montuori, 2011; Glǎveanu et al., 2019). Fostering creativity and expert performance in sport and physical activity is often predicated on new training paradigms that provide novel performance insights and develop innovative practices. The Fosbury flop is an example that quite literally raised the bar (Orth et al., 2017) and redefined human performance and potential in the high jump event.

The example of the Fosbury flop in sport suggests that non-linear transitions in performance could be supported by contemporary theorizing on teaching and coaching to foster creative development and realize human potential across the lifespan. This paper offers a new point of departure for research aimed at practical applications in sport and physical activity. Initially, we argue that developing creativity is a multifaceted challenge requiring research approaches that incorporate, rather than isolate, interdependencies and interactions between athletes and their environment. Next, we outline the conceptual adjacency of key concepts from transdisciplinarity, dynamic systems theory, ecological psychology and socio-cognitive psychology in order to extend the scope of ecological dynamics, better contextualize the application of non-linear pedagogy and illuminate our view of creativity in sport. Finally, we discuss the role of sociocultural constraints on creativity by undertaking a contextual analysis and offering practical considerations.

More than a mere performative act we conceptualize creativity as a crucial component of human development and recognize its relevance for all sports participants at all levels (Rasmussen et al., 2017). Creativity is not only a point to reach; it is the process or means of reaching for human potential. It is a development resource that inspires further learning, especially in sport (Rasmussen et al., 2017). A majority of creativity research in team sport has focused on a tactical emphasis founded in information processing models of cognition (Furley and Memmert, 2015; Memmert, 2015). However, broadening perspectives are shifting toward a recognition of athlete-environment dynamics (Hristovski et al., 2012) and exploring: the role of early life social interactions (Martin and Cox, 2016), affordances (Glǎveanu, 2012; Rasmussen et al., 2017), movement variability adapting to constraints (Orth et al., 2017) and multidisciplinary frameworks of creative development (Santos et al., 2016).

The challenge of developing and enhancing creativity in the modern world is a complex one. Countless interactions between people and places, influence myriad development processes impacting multiple aspects of creativity. In emphasizing the sociocultural basis of understanding creativity, a recent commentary highlighted that the creative process needs to be conceptualized as embodied, multi-dimensional, culturally-mediated, relational, dynamic and situated, amongst other facets (Glǎveanu et al., 2019). To better comprehend the *inter-relatedness* of ecological and human sub-systems there have been increasing calls for transdisciplinary approaches (Balagué et al., 2017). We contend that this type of approach could highlight the sociocultural and historical constraints on creative development and demonstrate the pursuit of human potential as a *wicked problem* (Levin et al., 2010).

### Wicked Problems and Developing Creativity

Wicked problems are global challenges that often involve many societal groups and social systems, have unpredictable consequences, and do not lend themselves to straightforward, traditional solutions (Rittel and Webber, 1973). To exemplify, consider the multifaceted influence of corporate capitalism, not only is it an economic system that exploits the worlds material environment (Brown et al., 2010), but recent research points to a toxic influence on sociocultural systems as well. Unrestrained advertising has been found to foster harmful social conditions and contexts that are detrimental to psychological development, impacting human potential, particularly during childhood (see Kasser and Linn, 2016). The *wickedness* of interrelated problems is not only about high degrees of complexity, but also represents a recognition that no level of linear (or mono-disciplinary) thinking might present a workable solution. Critically, the wickedness requires a holistic, deeply contextualized understanding of problems and positions solution generation as a secondary concern (Plamer et al., 2007).

Creative problem solving has been called upon to address the world's wicked problems (Songca, 2006; Bocchi et al., 2014). However, cultivating the cultural contexts and social systems that foster human creativity requires that we confront the challenge of developing creativity, a wicked problem in its own right. As a brief example of this wickedness, consider that the dominant “understanding of creativity has been shaped by cultural, methodological, and epistemological factors” (Montuori and Purser, 1997, p. 1) and that the majority of studies of creativity in the literature originate from cultures that orientate strongly toward individualism, and whose scientific approaches have typically been reductionist (Montuori and Purser, 1997; Glǎveanu et al., 2019). “If creativity is viewed as *nothing but* a factor of personality, or *nothing but* a factor of cognitive or genetic forces, or, for that matter *nothing but* the product of historical forces, then we are falling victims to a kind of reductionism (whether genetic, psychological, or sociological) that severely restricts and impoverishes our understanding of creativity” (Montuori and Purser, 1997, 14 italics in original). In this paper we aim to cultivate a more holistic understanding of creative development by demonstrating that fostering creativity and human potential, like many of the world's complex and ill-defined challenges, is a *wicked problem* (Levin et al., 2010) in need of transdisciplinary inquiry (Songca, 2006).

### The Potential of Transdisciplinary Research

To better comprehend wicked problems and transcend traditional linear thinking in sport, work organization and education there have been increasing calls for transdisciplinary research (Songca, 2006; Bocchi et al., 2014). At present, transdisciplinary research is flourishing in the field of sustainability science. In over 20 completed, or ongoing projects, transdisciplinary processes lay the foundations for investigations into the complex interactions of socio-ecological systems (see Herrero et al., 2018). In sport, transdisciplinary approaches are scarce. However, a recent study demonstrated potential benefits and revealed the sociocultural forces influencing Australian talent development programs (Toohey et al., 2018). Academics and practitioners across diverse theoretical perspectives came together to investigate four sports—Australian rules football, cricket, kayaking and tennis—and concluded that a complex composition of physical, psychological, environmental and contextual factors shape talent development (Toohey et al., 2018). To capture the complex interactions of these multiple facets to advance theoretical knowledge and improve applied practice, it is vital that sports research transcend mono-disciplinary approaches, and move beyond a “paradigmatic, quantitative (*often reductionist*), sport science lens” (Toohey et al., 2018, 356 italics added).

A sport science lens can be characterized by quantitative, typically reductionist and mono-disciplinary approaches to physiology, motor-learning, biomechanics, and psychology. This lens has shaped applied practices that range from: strength, conditioning and injury rehabilitation; coaching pedagogy; video analysis and much more. The reliance on a positivist lens creates bias toward a one-dimensional, decontextualized and cyclical production of fragmented knowledge (Alhadeff-Jones, 2009). As such, a majority of sport science research and practice has been limited by a history of “organismic asymmetry,” foregrounding the “internal mechanics” of the athlete and neglecting relations with the environment (for a detailed argument see Davids and Araújo, 2010). The separation of organism and environment, alongside other decontextualized (and mono-disciplinary) approaches “enormously simplifies the type of integration among organizational levels in living systems” that range “from genes to social systems” (Balagué et al., 2017, p. 2). That is, the *inter-relatedness* of sub-systems makes it challenging to understand (from methodological and theoretical perspectives) how different dimensions of human behavior (e.g., physical, cognitive, psychological, emotional, social, cultural, and historical) continuously interact to influence performance and development (and creative moments) on various timescales (Davids et al., 1994; Balagué et al., 2017). As a result, many practitioners, coaches and sporting organizations educated in the traditional “sport science lens” hold an (ontologically) limited picture of the complexity of sports performance and human development (Mallo, 2015). In practice, the design and assessment of training sessions becomes dominated by statistical analysis of “kilometers run” and “sprints made” (quantitative measures of physical load). This critique is not an assault on disciplinary research, or a call for sports science to abandon performance measurement and evaluation entirely. However, it spotlights the need to transcend the limitations of traditional biases in order to aid practitioners faced with real world, and increasingly wicked problems. Montuori (2013) described a practical limitation of mono-disciplinary research that is particularly poingient for sport:

Disciplinary approaches have historically produced some excellent research, but they are also limited and limiting…The problem is not that such research is not interesting or important per se, but that it gives us a partial view, and this view is often – despite warning labels – taken to be the whole. Using that partial view as a lens through which to view the entire phenomenon becomes problematic, particularly for practitioners. Unlike academic disciplines, life does not break down into neat categories and disciplines, and we ignore this at our own risk (p.46).

Both trans- and multidisciplinary approaches aim to alleviate the limitations of mono-disciplinary research and construct a more holistic, “workable” picture to aid practitioners. The creativity development framework is an admirable example that aids the transition toward a more holistic view of creative development in team sport (Santos et al., 2016). However, while multidisciplinarity represents an important step in the right direction it can remain susceptible to the mono-disciplinary limitations of its constituent parts. Unlike transdisciplinarity (elaborated upon below), the paradigmatic limitations of combined disciplines can remain in multidisciplinary approaches. A related issue concerns the inadvertent amalgamation of disparate fragments of decontextualized knowledge founded in distinct theoretical (or philosophical) perspectives with incongruent ontological and epistemological foundations (Sefotho, 2015).

In sport, multidisciplinary frameworks aiming to aid practitioners appear unconcerned or unaware of the intra-paradigmatic limitations of the “quantitative, sport science lens,” leaving processes, procedures, systems and practices in sport susceptible to “organismic asymmetry” (Dunwoody, 2006; Davids and Araújo, 2010). This bias displays limited recognition of the inter-relatedness of human and ecological sub-systems (Balagué et al., 2017) and the sociocultural forces (Toohey et al., 2018) that characterize environmentally situated constraints emerging from sociocultural historic macro contexts (Rothwell et al., 2018, 2019) (detailed in the following sections). Such oversight facilitates a linear picture of human development that contradicts the everyday contexts and unpredictable situations that sport practitioners find themselves in (Bowes and Jones, 2006). In the context of this paper, a major concern with approaches to mono- and multidisciplinary sport science is that they fail to address the implications for practice of the rich complexity of social systems (Alhadeff-Jones, 2009). This limitation fails to comprehend the socially and culturally embedded person-environment interactions at the core of human development and creative moments (Glǎveanu, 2012; Eisler et al., 2016; Glǎveanu et al., 2019). This current weakness in sport science could be remedied by practitioners adopting a transdisciplinary platform to underpin their work. Sports development frameworks aimed at practice should emerge from, and evolve in, interaction with the sociocultural context in which practitioners are embedded. This embeddedness will ensure that any framework is inherently contextualized and co-created from the bottom up as much as the top down. This is the promise of transdisciplinarity, because in contrast to mono-, multi-, and interdisciplinary approaches, transdisciplinary inquiry foregrounds the need for a reciprocal *top down, bottom up dialectic* (Songca, 2006) between academics, practitioners *and* athletes.

At the heart of a transdisciplinary approach is the recognition that no one academic discipline can provide appropriate methods and data to illuminate the inter-relatedness of a particular issue (Songca, 2006). Instead, transdisciplinarity challenges researchers to better appreciate the indivisible interconnections between our biological and social systems (Balagué et al., 2017) and overcome a culture of intra- and inter- disciplinary competition (Songca, 2006). Ideas acknowledged by Poincaré (the founding father of dynamical systems theory), who noted that science is a system of relations, and that it is in relations alone that objectivity must be sought and, consequently, that “mathematicians do not study objects but the relations between objects…” (Poincaré, 1905, p. 20; translation by George Halstead, 1907).

Compared to traditional (mono-disciplinary) approaches, transdisciplinary inquiry is characterized by four defining features (Montuori, 2013). Specifically, it: (a) is inquiry-based rather than discipline-based, (b) integrates rather than eliminates the inquirer from the inquiry, (c) adopts a meta-paradigmatic rather than intra-paradigmatic approach, and (d), utilizes complex systems theorizing and embraces complexity rather than a reductionist perspective (Montuori, 2013). Transdisciplinary inquiry can be thought of as an *undisciplined approach*, inspiring research projects that balance “methodological groundedness and epistemological agility” (Haider et al., 2018, p. 191). Developing a generative space, transdisciplinarity can utilize methodologies that view phenomena from other, broader, and more holistic perspectives and embrace systems with multiple ontologies (i.e., order, complexity and chaos) and nuanced interactive relationships between system components (Snowden and Stanbridge, 2004). A transdisciplinary approach is particularly generative for appreciating the sociocultural conceptualization of creativity (Glǎveanu et al., 2019) within complex and dynamic sport performance environments (Araújo et al., 2006). While it is by no means a panacea for wicked problems, or a silver bullet to avoid historical bias or epistemological hegemony, transdisciplinary inquiry provides a more ontologically accurate, and generative, point of departure to understand a particular wicked problem; that is, developing creativity and reaching our human potential, in sport and life.

### Dynamic Systems Theory: A Transdisciplinary Foundation

Recognizing the need for transdisciplinary inquiry, in this paper we: (a) adopt the broad scope of inquiry needed to comprehend creative development; (b) highlight knowledge as an embedded web of interconnected relations (Montuori, 2013); and (c) embrace dynamical systems theory as a meta-paradigmatic approach. Our approach signifies that general concepts of dynamic systems theory are valid for all levels of analysis, from molecules to social systems (Balagué et al., 2017). We aim to analyze key issues and critique outdated paradigms while synthesizing novel concepts from ecological dynamics and ecological psychology alongside established concepts from socio-cognitive psychology. At the core of this conceptual analysis, we argue for a re-conceptualization of the concept of human creativity to emphasize the interaction of social, cultural, historic, and relational dimensions on achieving potential and expertise (Eisler et al., 2016). This perspective suggests that the very essence of creativity “is one of connection and/or interdependence rather than abstractions and/or independence” (Eisler et al., 2016, p. 17). To explain these perspectives in the sporting realm we utilize the theoretical framework of ecological dynamics (Araújo et al., 2006).

### Ecological Dynamics

The seeds of ecological dynamics emerged from the work of Davids et al. (2001), Beek et al. (2003), and Warren (2006). These authors started to recognize the conceptual adjacency of key concepts and tools in dynamical systems theory and ecological psychology applied to sport. Ecological dynamics as a theoretical framework (Araújo et al., 2006) was proposed to build on these original attempts. This framework also integrated key insights from the complexity sciences, evolutionary sciences and constraints theory (Newell, 1986; Kauffman, 1996), especially emergence and system self-organization under interacting constraints, to explain human movement behavior and moments of creativity within sport performance and motor learning contexts.

Within ecological dynamics, non-linear interactions between system components provide a framework for understanding the exploratory fluency and flexibility of adaptive and creative movement solutions (Hristovski et al., 2012). Fluency is considered the generation of numerous, functional performance solutions (Seifert et al., 2014) and flexibility refers to the variety of class solutions (Kim, 2006). Within fluid athlete-environment systems, we propose that moments of creative movement are emergent and arise from a combination of dynamic constraints. Specific moments of creative movement can be conceptualized as emergent behavioral tendencies arising under specific constraints, which elicit adaptive actions. The implication is that specific constraints (i.e., task or sociocultural) can be manipulated and exploited in practice designs to provide opportunities or invitations (affordances, detailed later) for creative behaviors to emerge from athletes. In the following sections, we aim to expand on this position and develop the ecological dynamics rationale by clarifying the following proposal: cultivating a constellation of constraints within a context, culture or *form of life* (Wittgenstein, 1953; Rietveld and Kiverstein, 2014) can facilitate creative development. In particular, we discuss how complex system and transdisciplinary approaches resonate with conceptual frameworks that recognize the irreducible embeddedness of an athlete and a sport environment (Gibson, 1966; Bronfenbrenner, 1979; Henriksen et al., 2010; Sheldon et al., 2011; Mahoney et al., 2014; Henriksen and Stambulova, 2017). We propose that sport “teams and individuals, are systems that face ill-defined problems” (Hristovski et al., 2012, p. 26) and that new models of pedagogical practice should be predicated on notions of athletes and sports teams as complex adaptive systems (Chow et al., 2007; Davids, 2015; Silva et al., 2016).

## A human Ecology of Complex Adaptive Systems

**Figure 1:**
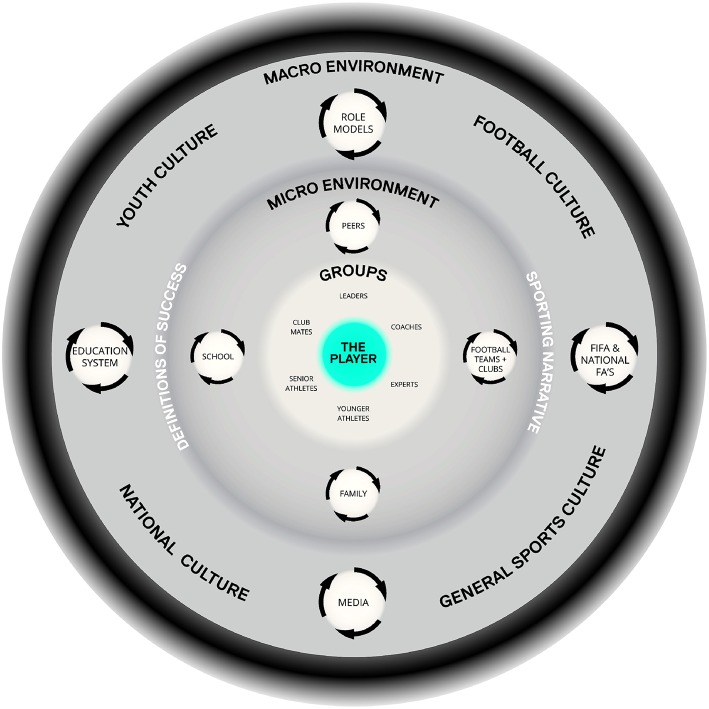
Illustrates the ecological context of football player development. Originally adapted from “Athlete Talent Development Environment,” by Henriksen et al. (2010), reproduced here with permissions from the Player Development Project Copyright 2017.

Gibson's (1966) approach to ecological psychology emphasized the interdependence of an organism and its environment (Araújo and Davids, 2009). Applying Gibson's approach to human development, Bronfenbrenner's (1979, 1995, 2005) *bioecological model* provides a reference point to understand the athlete-environment relationship as a particular ecological niche (or human ecosystem). Bronfenbrenner's model suggests humanistic and psychological needs are satisfied, allowing psychosocial skills to evolve, through a person's embeddedness within particular sociocultural contexts (Larsen et al., 2013). Holistic life skills and psychological constructs (e.g., identity, mindset, and motivational profile) evolve while people are embedded (i.e., contextualized) within different environments (McAdams, 1995). The multi-level personality in context model (Sheldon et al., 2011) highlights an individual's social-entrenchment as a fundamental influence on the satisfaction of basic psychological needs (Deci and Ryan, 1985). Variability in satisfaction of basic psychological needs might facilitate or thwart many development processes, including: (a) enculturation (Crotty, 1998); (b) acculturation (Kim and Omizo, 2003; Sam and Berry, 2010); (c) enskillment (Ingold, 2000); (d) internalization of behaviors (Deci and Ryan, 2000); (e) assimilation of values (Deci and Ryan, 2000); and (f), humans encompassing natural growth tendencies (Vansteenkiste et al., 2006). System, multilevel, and/or ecological approaches to human development provide complementary frameworks to comprehend the complex human sub-systems and development processes that are constantly modified by our experiences and relationship with the surrounding environment (Bowes and Jones, 2006; Montuori, 2012). These concepts are beginning to be applied to athlete development. For example, Henriksen et al. (2010) adapted Bronfenbrenner's bioecological model (Bronfenbrenner, 1979) and defined the athlete talent development environment (ATDE: see Figure 1 for an example), as:

… a dynamic system comprising (a) an athlete's immediate surroundings at the micro level where athletic and personal development take place, (b) the interrelations between these surroundings, (c) at the macrolevel, the larger context in which these surroundings are embedded, and (d) the organizational culture of the sports club or team, which is an integrative factor of the ATDE's effectiveness in helping young talented athletes to develop into senior elite athletes (p. 160).

More recently the holistic ecological approach (Henriksen et al., 2010; Henriksen and Stambulova, 2017) has been applied to football player development (Larsen et al., 2013, 2014) and adapted into a football specific athlete talent development environment (Figure 1). This holistic picture of player development (Figure 1) is currently guiding research and education practices at AIK Football Club, Stockholm, Sweden (Vaughan et al., 2017).

Considering each athlete-environment relationship (Figure 1) as an interaction between complex adaptive systems requires an appreciation that individuals and environments co-exist as open, dynamic, and nested systems. Open systems, in contrast to closed systems, are capable of exchanging energy and information within the surrounding ecology (i.e., at a macro and micro scale) (Von Bertalanffy, 1950; Ashby, 1956). It is from the interactions of such complex adaptive subsystems—neurobiological, psychosocial, socio-cognitive—that creative behaviors might materialize, emerging through the dynamic, fluctuating relationship between the individuals and their environment (Davids et al., 2013). Montuori (2011) suggested that once appropriate fluctuation (i.e., disequilibrium) is created by increasing system complexity, a critical (bifurcation) point is reached. At this moment, the system can move in multiple directions, due to the system property of metastability (Kelso, 2012; Passos et al., 2016) and if a different state of self-organization (e.g., a potentially creative behavior) does not emerge, the system returns to a previous state. It is proposed that transdisciplinary knowledge is needed to understand the emergence of behaviors in self-organizing systems.

One characteristic of living (open) systems that helps conceptualize the extent of information exchange with a performance environment is disequilibrium. The more open the system, the greater the opportunities for exchanges, interactions, and exposure to experiences of difference, novelty, complexity, and functional disequilibrium (Montuori, 2012). As one example, the more open the athlete-environment system, the greater the movement variability available to support adaptations, and the greater the potential for creative moments (Orth et al., 2017). In cognitive psychology, the suggestion that a wide breadth (openness) of attention (i.e., perception, attentional style or focus) is facilitative for creative performance (Carson et al., 2003; Friedman et al., 2003; Healey and Rucklidge, 2005; Hristovski et al., 2011) might give insight into an aspect of openness of actions, perception and cognitions. In an ecological dynamics rationale of skilled movement and coaching praxis, athlete openness for interactions within a performance environment is considered as attunement and utilization of affordances.

### Affordances, Exploratory Behavior, and Creative Moments

Affordances are opportunities or invitations (Gibson, 1979; Withagen et al., 2012) for actions that emerge as athletes interact with critical information from the environment (Travassos et al., 2012). Within ecological psychology, information can be perceived as relational and influenced by the specific intentions of the athlete and opportunities to act within the environment (van Dijk and Rietveld, 2017). Therefore, affordances emerge when lawfully-specified possibilities and athlete intentionality (i.e., goal directed behavior) are coordinated (Turvey, 1990). When “perception is of affordances (opportunities for action)” (Araújo et al., 2017, p. 10) then “behavior can be understood as self-organized under constraints” (Araújo et al., 2017, p. 13). The organization of an athletes' behavior is not imposed from inside (e.g., the mind) or outside (e.g., reinforcement contingencies, or coach instruction) but emerges in a constant dynamic with the performance environment (Araújo et al., 2017). Providing a departure from traditional approaches to creativity, and aligned with these ideas Orth et al. (2017) rejected the idea that “individuals first generate an idea in their mind, which is then enacted in behavior” (2). Supporting the concept of behavior as self-organized under constraints Orth et al. (2017) clarify that, “rather than referring to ideas that are uniquely generated by a (creative) cognitive system, we use the term creative as a descriptive for unfolding actions that are original (relative to the individual or group) and functional (i.e., they support task success)” (2 italics in original). Therefore, the design of tranining environments and manipulation of constraints becomes focal for practioners aiming to foster exploratory behavior and creative moments.

Exploratory behaviors emerge from the metastable dynamics of system self-organization. It is within the self-organization of intertwined action, cognition, and perception processes that constraints impinge upon these systems and influence our movement behavior (Hristovski et al., 2012). Creative moments might arise from the idiosyncratic configurations and interactions of systems continually shaped by the sociocultural, personal, environmental, and task constraints in each athlete-environment interaction (Hristovski et al., 2012). In sport contexts, the interdependence of affordances and effectivities (individual action capabilities) helps to capture the relationship between performer constraints and elements of the environment (Fajen et al., 2009; Lopez-Felip and Turvey, 2017). From the perspective of developmental psychology, learning about affordances requires children to explore novel activities which might expand their effectivities and diversify skills (Gibson, 1988). Exploratory activities involve the sampling of varied movement opportunities—sport, dance, martial arts and general play (Côté and Ericsson, 2015; Davids et al., 2017)—available within a *form of life* (introduced below). As a child's perceptual systems develop, exploratory activities are used to discover affordances relevant to their current stage of development. In other words, “as new action systems mature, new affordances open up and new ‘experiments on the world' can be undertaken” Dependent upon their stage of development and experience, each athlete will be open to certain affordances and closed to others in the landscape. To exemplify, in football, analysis of competitive performance at under 17 and senior professional levels, may reveal that the there is less time and space to shoot at a senior level. The implication is clear for the design of training sessions for the younger age group. Athletes of varying developmental capacities might be assigned different *challenge points* (less time and space progressively) in a shooting practice and work through different shooting tasks at their own pace, sampling specific actions and exploring their creative potential for creating goal-scoring opportunities (Santos et al., 2016) by concurrently expanding effectivities and discovering affordances. The emergence and gradual acceptance of a “toe poke” finish (scoring shot) in football is a relevant case. A legitimate skill in futsal (5-a-side indoor football, whereby time and space are reduced) the “toe poke” was initially ostracized by many in football. However, a number of high profile goals (Maradona, World Cup 1986; Romário, World Cup 1994; Ronaldo, World Cup 2002; Bradley, World Cup 2014; Oscar, World Cup 2014, see FIFATV, 2015) have been scored with goalkeepers seemingly deceived by the speed and lack of “back lift” when shooting. It is possible that futsal experience in developing athletes may enrich their skill sets so that there is potential to exploit a toe-poke to deceive defenders and a goalkeeper when there is restricted time and space in the penalty area in association football. Furthering understanding of how creative potential for specific actions might be explored in practice, Hristovski et al. (2012) identified how small manipulations of a task constraint (e.g., scaled distance of an athlete to a punching bag) resulted in variations of exploratory breadth, fluency, flexibility and originality of movement behavior (punches). The affordance landscape confronting the trainee boxers was significantly changed with variations in scaled distance to the punch bag, resulting in different values of scaled distance affording the emergence of different hitting actions.

Recent studies within multiple team sports (e.g., Headrick et al., 2012; Orth et al., 2014) have demonstrated how decision making and the coordination of action are adapted to changing task constraints that alter the detection of critical information: “Critical information sources (i.e., affordances and action capabilities) continuously shape intentions and enhance decision-making, planning and organization, during goal-directed activity” (Davids et al., 2013, p. 23). These ideas suggest that as expertise develops, the detection of critical information could be progressively scaffolded by task constraints that promote (potentially novel) critical information sources. For example, football players might become attuned to gaps between defenders, then spaces emerging between lines of defenders, and perhaps even the weight distribution (knee and hip angles) of a defender when attempting to dribble past them in a football match. Such information might prove critical in discerning the balance of a defender, and light reflected from these body parts can provide optical information for an opportunity/affordance to dribble.

### A Non-linear Pedagogical Framework

Founded in the ecological dynamics rational, non-linear pedagogy aids practitioners in attempting to scaffold athlete attunement to critical information in a performance environment. The pillars of non-linear pedagogy are “pedagogical principles that emphasize representative learning designs; manipulation of task constraints; infusion of movement variability; establishing close information–movement couplings; and the modification of attentional focus instructions” (Chow et al., 2016, p. 61). We propose that by first identifying and then manipulating (sociocultural, task, and environmental) constraints on learning, sport coaches might be able to design practice environments that more readily afford opportunities for learners to explore functional performance solutions, develop skill and enhance their creativity. Such practice session design would support the self-organization and adaptability of athlete behavior in relation to changing constraints in the sporting environment (Esteves et al., 2011). An example of football practice session design and constraint manipulation founded on the principles of non-linear pedagogy can be seen in Video 1.

This type of pedagogical approach aims to avoid the traditional tendency in sport to rehearse and reproduce specific actions proposed by a teacher or coach (Chow et al., 2016). “Within the framework of non-linear pedagogy, a skill and more generally talent, is not a trait possessed by individuals alone but a property of the athlete-environment system subject to changing constraints” (Hristovski et al., 2012, p. 27; see also Davids and Araújo, 2019). An important point to note is that task, environment, and performer constraints are fundamentally influenced by sociocultural constraints (Araújo et al., 2010; Araújo and Davids, 2011; Hristovski et al., 2011). Due to the sociocultural embeddedness of the interactions between task, athlete, and environmental constraints, experience differs in myriad ways. Each individual might perceive and experience the performance landscape (shaped by many influences, including interdependence of affordances and basic psychological need satisfaction) differently and, therefore, engage in differential exploratory (potentially creative) behavior as a result of their unique athlete-environment system (Hristovski et al., 2012). Therefore, there is the need to recognize that any application of principles of non-linear pedagogy takes place in a sociocultural context, and that this context, plays its role in affordance emergence.

### A Form of Life, Affordances, and Football Playing Styles

Rietveld and Kiverstein (2014) have suggested that affordances are dependent on the skills available within a particular ecological niche, sociocultural context or wider macrosystem (for an illustration see Figure 1). Due to their relational nature, the variety of affordances are as rich and varied as the abilities (i.e., skills) socialized by sociocultural practices (i.e., enculturated) (Ingold, 2000). These authors have argued that it is the availability of sociocultural practices (constituted by beliefs, skills, habits, customs, attitudes) that defines a dominant *form of life* or way of doing things (Rietveld and Kiverstein, 2014). A form of life (Wittgenstein, 1953) can be described as regular behavioral patterns (e.g., movements, ways of perceiving or otherwise) manifest as sociocultural-environmental constraints on the normative behaviors and customs of our communities and cultures (van Dijk and Rietveld, 2017). The relevance is furthered when we appreciate that each sporting context is contained within its own form of life, which will facilitate engagement with some affordances, while limiting interactions with others. For example, compare the opportunities to dance in England and Brazil; will an English or Brazilian form of life (cultural context) afford football players more or fewer opportunities to explore movement potential through dance (Uehara et al., 2018)?

The openness to, and discovery of, novel affordances allows the application and diversification of skill within different aspects of the environment, creating potential novel movement patterns or forms of creative behavior (Rietveld and Kiverstein, 2014). This is perhaps best depicted in football by the (historical) Brazilian style of play and particularly evident in football players exposed to the acculturation and skilling of samba and capoeira, alongside the assimilation of *ginga* and *malandragem* (a culturally endorsed value characterized by cunning, street smarts and trickery) (Uehara et al., 2018). Providing a supportive rationale for the influence of samba and capoeira on creative development, Rietveld and Kiverstein (2014) stated, “by acquiring abilities that flourish in different sociocultural practices than one's own [outside of football], one can come to see new possibilities for action provided by the material environment [when playing football]” (327, words in brackets our addition).

Exploring the relational nature of affordances is essential for creative (movement) professions (Rietveld and Kiverstein, 2014) because it places further emphasis on the need to understand and explore the interaction between the openness of both the athlete and their environment. The openness of the human perceptual system (a sub-system of the athlete) and the openness of the form of life and playing style (systems related to the environment), might become critical interdependent factors in fostering creativity. From this perspective, we propose that it might be beneficial to zoom in and out on the athlete-playing style system and conceptualize it as relevant field of affordances within a form of life (van Dijk and Rietveld, 2017.) In order to do so, it is important that we can justify the playing style as an interdependent manifestation of the wider environment; a network of interdependencies that includes constraints emerging from the macrosystem and other sociocultural contexts and practices (Rossing and Skrubbeltrang, 2016).

Within team sport, the dominant form of life might be conceptualized as the deeply acculturated, socially accepted, and often taken for granted (nationally or regionally or trending) *playing style*. For example, affordances for football interactions, such as risking ball possession with long balls or maintaining ball possession with short passing, will be constrained by the (many interdependent) influences that shape a dominant playing style. Here it is proposed that a playing style is an emergent sociocultural artifact (Rossing and Skrubbeltrang, 2016), which embodies the manifestation of the relational environment when playing football. As an example, consider the case of F.C. Barcelona: In recent history the club has exhibited a style of play that often aims to maximize passing opportunities as affordances to maintain ball possession while simultaneously creating the space needed to score goals. As part of a development process, F.C. Barcelona academy players become attuned to the width of playing areas and simultaneously co-create a varied range of passing affordances which continuously emerge and decay from the co-creation and sharing of space in learning designs. F.C. Barcelona exemplify the hypothesis that the sociocultural context (form of life) influences training session design, player attunement and playing style. The Catalan culture is renowned for an *egalitarian passion for width* which manifests in the co-creation and sharing of space, particularly evident in seventeenth century Catalan gothic church architecture (Hughes, 1992).

Playing styles, both team and individual, emerge from myriad transdisciplinary constraints. These include but are not limited to (a) self-organizing degrees of freedom in a complex adaptive system; (b) the culturally defined task (challenge or goal); (c) dynamic affordance landscapes; (d) socio-cognitive motivational climates; and (e), sociocultural values and narratives. Too often, dominant playing styles tend to reinforce movement reproduction and conformity, limit engagement with exploratory affordances and restrict deviation from prescribed behaviors (Hristovski et al., 2012). Constraining movement behavior, in this way, will limit an athlete's capacity to become progressively attuned to information that specifies the rich abundance of affordances within a particular sporting environment (Rietveld and Kiverstein, 2014), thus limiting athletic potential.

### Sociocultural Constraints

Coaches working within one cultural context too often focus on the content—*the what of coaching* (session plan, game model, tactical plan)—taking for granted the culturally constituted methods of delivery—*the how of coaching* (Stambulova and Ryba, 2014). Recognizing coaches as agents working within complex adaptive systems (Bowes and Jones, 2006), subject to sociocultural constraints, reinforces the need to reflect on the contextualized, culturally sensitive *how of coaching*. *What* coaches direct athlete attunement toward (i.e., awareness of spaces, gaps, passing opportunities, defenders balance) and *how* (i.e., pedagogy; session design, questioning, and co-creating) attunement is directed is subject to the sociocultural constraints (perhaps representing the subconscious *why*) that shape group cultures, forms of life and playing styles. Coulter et al. (2016) highlight how sociocultural constraints might emerge to shape playing style when explaining problem solving within a group culture. They suggested that if a group (i.e., team) problem is solved repeatedly, in the same way, the solution (e.g., a pass/dribble etc.) becomes an evident truth accepted within the group culture by those group members. When accepted as an evident truth, this solution can become a sociocultural constraint that influences future problem solving and movement behavior of group members (players) and future group members. Sociocultural constraints might be considered as an explanation for the emergence of traditional, distinct playing styles. For example, Brazilian football players and teams were once characterized (stereotyped?) by flair, creativity, and flamboyant dribbling originating from and reinforcing their own form of life and playing style; while the historical British Isles' approaches to football have often been characterized (stereotyped?) by direct long balls, hard tackles and risk-averse football, typically shaped by perceptions of rationality and hyper-masculinity in traditional forms of life.

Sociocultural constraints exemplify that “humans adapt to their social-ecological surroundings in complex ways” and support the rationale “that people's enduring cognitive structures, including values, are part of that adaptive process” (Manfredo et al., 2017, p. 776). When investigating values as constraints on perception-action, Hodges and Baron (1992) suggested that “ontologically, values are global constraints on an ecosystem” (270). They provide the formative conditions for the ecology while also shaping its dynamics and constraining the emergence of new ontological, epistemological or ethical features. “Thus, values are the *intentions of the world as a self-organizing system* in the sense that they are the ends toward which the ecosystem as a whole is directed” (Hodges and Baron, 1992, 270 italics in original). Critically, Hodges and Baron (1992) stated that “without the *higher-order constraints* we have called values, we think such an analysis of perception-action cycles, particularly in a sociocultural context, will remain enigmatic” (270 italics in original). In F.C. Barcelona (micro environment), an *egalitarian passion for width* might represent a key value and sociocultural constraint (emergent in the macro context) constituting, constraining and reinforcing the clubs unique playing style. A playing style (or football ecological niche) in which players perceptual systems and effectivities develop in interaction with an intention to share space and create a diverse range of passing opportunities (i.e., affordances), manifest from the egalitarian passion for width.

### Toward Practical Considerations

Within sport, we ought to consider the socializing influence of clichés, coach instruction, and fan feedback (resulting in the UK, in shouted commands like “get stuck in,” “second ball,” “mark space,” “play simple,” “attack,” “dominate”), especially as they relate to playing styles (termed an organization's “DNA”) and player intentionality (i.e., goal directed behaviors). It becomes particularly appropriate to appreciate *what* critical information is privileged by such communication and *how*—in its delivery, by coaches or significant others—communication influences the extent of athlete attunement to (critical) information sources. *What* critical information an athlete attends to might determine access to affordances within each ecological niche (form of life/playing style). Currently, athlete openness to affordances (and therefore opportunities for creative movement) might be constrained by the abilities (effectivities or skill sets) of those within their ecological niche; form of life; playing style; team; club; culture; nation; global sporting community—many nested systems. From an objective perspective, Rietveld and Kiverstein (2014) stated that affordances are more extensive than standardly recognized. Recognizing the myriad range of possible movement opportunities afforded to football players Lopez-Felip and Turvey (2017) describe the interdependence of the player-environment system as one whereby the system “constituents are non-denumerable and its semantics is functional, dependent on ‘when,' ‘where,' and ‘who.' It is a time-dependent and context-dependent semantics” (Lopez-Felip and Turvey, 2017, p. 169). Lopez-Felip and Turvey (2017) illuminated affordance-effectivity interdependence as reciprocal dispositional properties, such that when an organism is conjoined with its environment, the (re)organization of the system degrees of freedom emerges based on the commensurability between the particular kind of physical disposition (*affordance*) and the properties of the organism (*effectivities*) (Turvey, 1992). “Situation X affords activity Y for organism Z on occasion O if and only if X and Z are mutually compatible on dimensions of relevance to Y. Organism Z effects activity Y in situation X on occasion O if and only if Z and X are mutually compatible on dimensions of relevance to Y” (169). Creativity plays its physical role within this myriad arrangement of degrees of freedom and hints at the underlying thermodynamic processes when higher order states emerge from such non-equilibrium self-organizing systems.

The complex, functional semantics at the core of team sports might pose coaching itself as a wicked challenge. Knowing (or believing that we “coaches” know) *what* an athlete should be attuned to (critical information sources) might guide learning and or suppress creativity by creating conformity toward a perceived “ideal” performance model or technique (Hristovski et al., 2011). Rather, the suggestion is that *what* an athlete becomes attuned to (i.e., changing spaces, teammate and opposition movements, opportunities for football interactions) within a particular playing style, affords different opportunities for fulfilling human potential through movement, skill, and creative development. For example, I may perceive (be attuned to) affordances for direct long balls and remain unaware of concurrent opportunities to combine short, quick passes in potentially creative ways. This understanding is critical for coaches and other practitioners involved in continuously shaping (and re-shaping) the player development environment. The *what* of athlete attunement is critical in determining the information (relational nature of affordances) an athlete attends to and therefore the skill they develop; however, *how* athletes become attuned might hold potentially more influence in regard to ongoing engagement with, and exploration of affordances. Crucially, affordances only emerge to be engaged with if perception is intentional. Therefore, intentionality (i.e., goal directed behavior) and lawfully-specified possibilities must be coordinated for an affordance emergence (Turvey, 1990). van Dijk and Rietveld (2017) suggested that an individual manifests skilled intentionality in the context of their form of life (or playing style) by “considering the skillful responsiveness to multiple nesting and nested affordances simultaneously. i.e., the responsiveness to a whole *field of relevant affordances*” (van Dijk and Rietveld, 2017, 9 italics in original).

This is critical when considering *the how of coaching*; how do we design practice sessions that shine a light on key/nested affordances and utilize pedagogical strategies to foster an intentionality open to creative adaptation (see Video 1) rather than controlled by cultural clichés, authoritarian instructions, and performance models? For example, when coaches instruct players to pass wide or switch the play (as part of a performance or game model), they coerce behavioral outcomes rather than educate attention or attunement. Controlling commands aimed at specific behaviors inhibit the development of learners in achieving their potential. This prescriptive pedagogical methodology often disregards the critical information or lawfully specified possibilities (spaces, gaps, teammate, and opposition dynamics) that coordinate with intentionality of individual learners (e.g., maintain ball possession and find the time and space to score a goal) to form the affordances that invite movement. The risk is that instructional/coercive/controlling coaching results in attentional fixation and limits one's ability to remain open to critical information available in performance environments. It is recommended that future research explore the interdependence between player intentions and the form of life they are embedded or acculturated within. Transdisciplinary inquiry incorporating ethnography and guided by the skilled intentionality framework might prove generative in this area (van Dijk and Rietveld, 2017).

## Creative Development is a Wicked Problem: A Contextual Analysis

The importance of contextual/cultural sensitivity (as highlighted in the sections above) cannot be understated when tackling wicked problems and undertaking transdisciplinary inquiry; fundamentally “transdisciplinarity requires us to question values and cultures that were transmitted unconsciously during our professional training” (Songca, 2006, p. 227). A contextual analysis is a productive approach for investigating the sociocultural contexts in which phenomena are historically constructed (Uehara et al., 2018). The following contextual analysis demonstrates a transdisciplinary point of departure and introduces literature that illuminates some social, cultural, and historic constraints on creative development (Hristovski et al., 2012), and transdisciplinary research (Bocchi et al., 2014). To locate this contextual analysis we “zoom out” to appreciate the all-encompassing ecological context called the macrosystem. Of which Bronfenbrenner (2005) said “the macrosystem may be thought of as a societal blueprint for a particular culture, subculture, or other broader social context” (150) containing “the overarching pattern of micro-, meso-, and exosystem characteristics of a given culture, subculture or other broader social context” (cited in Uehara et al., 2014, p. 8). Critically, a macrosystem carries the information, ideology, and values that influence events and experiences at embedded levels. For example, the macrosystem influences contextually embedded microsystems—classrooms and coaching sessions—in which children develop (Kasser and Linn, 2016). We illustrated this perspective using the football specific example of an athlete talent development environment in Figure 1. The following analysis illuminates the wickedness of the challenge that constitutes developing creativity and reaching our potential due to macro level sociocultural constraints.

### Overly-Competitive and Comparative Contexts

Embracing transdisciplinarity and recognizing creativity as a collaborative interdependent endeavor (Eisler et al., 2016) requires critical reflection on the many societal systems (e.g., economic, media, educational, and sporting) that promote overly-competitive/comparative contexts (Kasser et al., 2007). Here, for example, it could be argued that wicked problems like climate crises (Kasser, 2009), dis-engaging educational programs (Taylor et al., 2008), and highly controlling sporting environments (Kidman and Lombardo, 2010) emerge from macrosystem constraints and the path dependency of what authors have termed “American corporate capitalism” (Kasser et al., 2007). Path dependency is a system process demonstrating that once a system is set on a development path, the historically derived (paradigmatic) modeling and emergent organizational structures constrain its trajectory (Djelic and Quack, 2007). American corporate capitalism (as opposed to Nordic, Asian and developing world versions of capitalism) is described as a dominate brand of capitalism that spread via globalization and had the largest worldwide influence at the turn of the millennium (Kasser et al., 2007). The ideology, values and path dependency of corporate capitalism continue to shape social contexts and organizational structures that over-emphasize individuality, competitiveness, hierarchy, and extrinsic rewards, often to the detriment of psychological wellbeing (Kasser and Linn, 2016) and at the expense of learning, creativity, and innovation in developmental and performative tasks.

### The Corporate Capitalist Macrosystem

Alongside toxic marketing and profit-driven educational philosophies, Kasser and Linn (2016) state that there are “many other ways in which the corporate capitalist macrosystem affects the exosystem structures and microsystem experiences that, in turn, influence children's development” (145). At the level of the individual, the psychological influences of corporate capitalism promote high materialistic value orientations that make people more likely to compete and less likely to collaborate (Sheldon et al., 2000). Over-emphasis on extrinsic rewards and competition is likely to reinforce social comparison, divisions between sub-groups, and insecurities, fear of failure, scarcity mentalities, and contexts that reproduce the controlling behaviors of teachers and coaches, currently evident in many educational environments (Taylor et al., 2008). An over-emphasis on internal and external competition has led to corporate organizational structures and governance practices being applied to elite and grassroots sport (e.g., professional football clubs and national governing bodies in sport) and education (schools and universities lumped together in national and international “league tables,” see Halffman and Radder, 2015). This approach might induce controlling (extrinsically rewarded) social contexts that fail to satisfy basic psychological needs, eroding intrinsic and self-determined forms of motivation that subsequently shape thoughts, affect, and behaviors (Deci and Ryan, 2000; Vansteenkiste et al., 2010). The problem with corporatist methods of working (e.g., Taylorism) is that they have been designed to encourage conformity and operational efficiencies in mechanistic processing systems (Myers and Davids, 1993; Rothwell et al., 2018). Such controlling management processes often (extrinsically) reward and reinforce individual competition and compliance at the expense of collective, potentially creative collaboration. Research has long suggested (Davids et al., 1991; Smith and Davids, 1992; Myers and Davids, 1993) that the reductionist, industrial paradigm, founded on the metrical regulation of Taylorism, might privilege a path dependency in education and training that focuses on (reductionist) *simplistic thinking*, in contrast to the contextualization and inter-connectivity that inspires *complexity thinking* (Bocchi et al., 2014).

### Conformity and Competition

According to Guilford (1959) problem solving occurs in diverse ways, through convergent and divergent thinking. However, education (on and off the field of sport) seems to mirror colonial/industrial/corporate traditions and focus on conformist, orderly, reproductive, and convergent thinking, which produces a reliance on same level, habitual, often pre-determined solutions. In comparison, divergent thinking fosters multiple solutions and was conceptualized as a foundation for creativity (Guilford, 1959). Montuori (2012) explains the controlling, reproductive, and overly mechanical nature of education: “Reproductive Education stresses conformity and homogeneity and suppresses creativity at a time when it is apparent that creativity needs to be mobilized to get beyond the decaying industrial views of modernity and envision new futures, new possibilities, new economic, environmental, social, and cultural and ethical systems” (65). To overcome the suppression of creativity, Barron (1990, 1995) has suggested that the benefits of complexity and variability (conceptualized here, as self-organization and disequilibrium) need to be better understood, promoted and recognized as conditions that inspire adaptive creativity. Recently, Bocchi et al. (2014) called upon educators to challenge the excessively individualistic view of creativity that, created by, and coupled with the conformist nature of many modern societies, has failed to account for the role of the environment in cultivating a generative space for ideas to flourish. Here they noted:

The problem is that our society is not designed for creativity but for machine conformity. Most importantly it does not support creative ideas. Let's just look at academia; having a good mind in academia means, among other things, to be razor sharp in critique. But we do not learn how to play with ideas, how to explore together, and support “newborn” ideas and allow them to flourish for a while. We immediately learn to attack and critique. In organizations, we laugh at “wild” ideas. We make jokes about people with their odd approaches. So as Oscar [Wilde] put it, art keeps people sane, but it is the environment that kills creativity in people, and arguably kills many creative people (Bocchi et al., 2014, 362 word in brackets our addition).

The work of Bocchi et al. (2014) reinforces the foregrounding of competition and backgrounding of collaboration in many modern societies, a dynamic that might thwart feelings of relatedness a basic psychological need (Ryan and Deci, 2000). Fostering emotional connectedness with others is an important characteristic for teammates in sport but also for theorists addressing wicked problems. The imbalance between competition and collaboration in scientific and coaching communities (see Potrac et al., 2013) might explain the process of *disciplinary purification* and the creation of intellectual silos (Montuori, 2013). Songca (2006) has suggested that: “Boundaries are drawn between the different disciplines and respect, recognition and promotions are acquired by publishing in areas that are related to one's discipline. The climate and culture that emanate from specific individual disciplines is one of competition and not collaboration” (p. 224).

A culture that underscores competing over co-operation keeps extra-disciplinary influences at bay, privileging orderly purity, despite arguments for complex inter- or cross- or even transdisciplinary work. When competition (and self-interest) dominates, educational systems are likely to focus on the extrinsic comparisons and generic measures that inhibit creativity, learning, and subsequent development.

### Path Dependency

In sports like professional football, a path dependency emerging from these influences might be exemplified by the “objective” and comparative measures of the English Premier League's elite player performance plan (EPPP) (English Premier League, 2011). The EPPP was developed using misconceived notions of a 10,000 h “performance clock” and appeared to reinforce many outdated machine metaphors mistakenly applied to development of human capacities such as expertise (Campitelli et al., 2015). Mechanistic approaches to coaching (for extensive critiques see Davids et al., 1998; Jones and Wallace, 2005) and reductionist sport science perspectives have been criticized for assuming that human behavior can be conceptualized as being “machine-like” and can be predicted and controlled in highly precise ways (Davids et al., 1991, 1994; Myers and Davids, 1993; Bowes and Jones, 2006).

In relation to creativity, it has been argued that the over-emphasis on competition, self-interest, and individualism spawned from macro constraints, like corporate capitalism, reinforces the myth of “the lone genius” (Montuori and Purser, 1995). This path dependent myth has monopolized the application of seminal creativity research by E. Paul Torrance (Kim, 2006). The Torrance tests of creative thinking have been largely (mis)used to identify *gifted* individuals, even though this was not the creator's intention. Here, Kim (2006) suggested that “Torrance's main focus was in understanding and nurturing qualities that help people express (*develop*) their creativity” (4 italics added). When administrating the tests, Torrance highlighted that environmental factors, like motivational conditions and psychological climate, would have an influence on peoples' creativity (Kim, 2006). However, this contextual line of research was not pursued and considerations of potential effects of motivational/psychological climates remain relatively underexplored. Montuori and Purser (1995) argue that the myth of “the lone genius” has prevented a comprehensive investigation into the social dimensions of creativity and the sociocultural constraints on creativity.

To summarize, we have aimed to illustrate how sociocultural constraints might emerge in interaction with macrosystem path dependency to inhibit the conditions necessary for creative development. As an example (at some levels of analysis) we have considered the tendency of corporate capitalism to foster an over-emphasis on competition rather than collaboration (Kasser et al., 2007). This imbalance might be considered a sociocultural constraint on creativity. Located at the level of the macrosystem, corporate capitalism might inhibit creative development by suppressing people's basic psychological need satisfaction and subsequent internal motivation experienced in the micro environments under its influence, such as workplaces and educational establishments including offices, classrooms, learning and training centers, and academies. Therefore, while calls to foster creativity in daily life (Runco, 2014), education (Robinson, 2009), and team sport (Memmert, 2015) are well-established, many organizational structures and pedagogical approaches remain fundamentally unsuited to fostering creativity due to these often unseen sociocultural and historical constraints.

In previous sections of this paper, we have argued that these constraints, which operate at a communal and societal levels, may be dominated by traditional *forms of life* imposed in bygone eras (see Rothwell et al., 2018, 2019). These forms of life shape the way that learning designs continue to be perceived in educational programs for trainers, teachers and coaches, exemplifying a type of “system capture” and inhibiting the adoption of new pedagogical models for enhancing human potential, innovation, and creative behaviors.

### Practical Considerations for Sport, Coaches, Clubs, Sports Organizations, and Conclusions

This conceptual analysis proposes that the first step toward nurturing creative moments (a wicked challenge) in sport, is cultivating an in depth understanding of culture and context alongside a nuanced appreciation of athlete-environment interdependence. We propose that conceptualizing a form of life and sociocultural constraints through the lens of ecological dynamics (Rothwell et al., 2018, 2019), represents a novel avenue in understanding athlete-environment interactions and creative moments. We contend that long-term athletic development and moment-to-moment creativity emerge from deeply contextualized athlete-environment interactions. Interactions that are shaped by, and subject to, a continually changing dynamic of constraints. Constraints that transcend disciplinary boundaries, act over varied timescales (Balagué et al., 2019), and can cascade from macro to micro environments. From this vantage point, practitioners might aim to design movement environments that encourage the discovery and exploration of novel affordances to better foster creative moments for achieving potential.

We propose that the skillful manipulation—dampening or amplifying—of sociocultural constraints on behaviors is central to developing an environment conducive to creativity. Manipulating constraints in the moment and over time (by coaches and other support personnel in football clubs, sports organizations, and governing bodies) can better co-create the environmental conditions, customs, habits, and ways of doing things, in a form of life that constrain and afford creative moments. However, this is by no means a straightforward endeavor, it is wicked challenge whereby sociocultural constraints are addressed at multiple levels. For example, consider a form of life and emergent playing style that promote an *over-emphasis on intra team competition* (often conducive to anxiety) leading to individualistic play. At the level of the micro environment a coach might aim to dampen this influence by designing training sessions with task constraints that require teamwork and collaboration. Concurrently, and at an organizational level, a sports club (or governing body) might aim to de-emphasize (the anxiety inducing) culture of competition between teammates, parents and coaches by removing *best-with-best* “academy” selection at young ages (a change implemented at AIK football club and supported by on-going transdisciplinary research) (Vaughan et al., 2017).

It is important to problematize the manipulation constraints in social systems at multiple levels, recognizing that outcomes are transient and unpredictable and that issues may shift and re-emerge due to the unpredictable consequences of wicked problems. As such, the ways in which sociocultural constraints may be manipulated differ from context to context. What is possible in Stockholm might not be possible in Barcelona. Equally, a club that suffers from anxiety inducing hyper-competition in some team contexts may contain certain players in particular teams who are overly concerned by “not standing out,” and or “being a good teammate.” Therefore, they require training sessions designed to encourage an exploration of individual solutions to tasks, with rules or constraints that, for example, enlighten dribbling opportunities. In the same club, one coach may be working with hyper-competitive “dribblers” while another coach is concerned that her team are overly-conformist “passers.” Here lies the wicked problem and the art of coaching: The point of departure when designing learning environments and cultivating contexts for creativity is a nuanced appreciation of sociocultural constraints that might be amplified or dampened to encourage athlete-environment interactions that foster creative moments in specific contexts. Therefore, what works in one context is often unhelpful in another. This is why it is crucial that any framework aiming to aid practice is flexible, contextualized and co-created from the bottom up as much as the top down. This is the promise of transdisciplinary endeavor, because it foregrounds the need for a reciprocal *top down, bottom up dialectic* (Songca, 2006) between academics, practitioners *and* athletes.

We suggest that future research seeks to explore the sociocultural constraints that continually influence our ecological niche and co-create a form or life, which shapes the available field of affordances needed for advancing potential through learning and development programs. Within the context of football player development, we suggest that a form of life might be conceptualized as habits, customs, beliefs, experiences, ways of doing things, and attitudes that create a climate/culture in a sport or sports organization. This holistic ecological perspective on skill acquisition encourages educational, pedagogical, coaching, and research approaches to adopt a transdisciplinary process of inquiry and aim to re-conceptualize creativity and human development as one of connection and interdependence within a surrounding ecology of complex adaptive systems. Transdisciplinary approaches guided by the skilled intentionality framework and ethnographic endeavor are recommended as avenues for future investigation (van Dijk and Rietveld, 2017). We also propose that some playing styles, by virtue of the affordances offered to learners and basic psychological need satisfaction in athlete development, might cultivate creative development and well-being more than others. In this way, transdisciplinary lines of inquiry might represent fertile ground for fundamental changes in the approaches we use to enhance learning and development of human creativity both on and off the field of sport.

## Author Contributions

JV carried out the drafting, conception, and design of the manuscript, wrote the general topics of the article, conceptualized the combination of theoretical models. CM contributed to drafting, conception, and design of the manuscript, ensuring that the ideas presented were appropriately investigated and articulated, critically revised the manuscript for important intellectual content. KD was involved in drafting the manuscript and ensured that the ideas presented were appropriately investigated and articulated with particular respect to ecological theories and system processes. PP revised the manuscript critically for important intellectual content, in particular revising the structure and providing analysis and interpretation of the sociological perspectives and coaching approaches. ML-F reviewed the manuscript for important intellectual content and revised sections articulating concepts from ecological psychology and football praxis.

### Conflict of Interest Statement

JV is employed by AIK Fotboll Stockholm and is a co-founder of Player Development Project. ML-F is employed by Futbol Club Barcelona. The remaining authors declare that the research was conducted in the absence of any commercial or financial relationships that could be construed as a potential conflict of interest.

## Supplementary Material

Video 1 Football training design founded on the principles of non-linear pedagogy.
